# The expected benefits of using social robots when working with institutionalised older adults: A qualitative study among healthcare professionals

**DOI:** 10.1371/journal.pone.0348438

**Published:** 2026-05-11

**Authors:** Sanja Balalic, Robbert Sanderman, Nick Degens, Marrit Tuinman, Mariët Hagedoorn

**Affiliations:** 1 School of Social Studies, Hanze University, Groningen, The Netherlands; 2 Department of Health Psychology, HPC FA12, University Medical Center Groningen, University of Groningen, Groningen, The Netherlands; 3 HKU University of the Arts, Utrecht, The Netherlands; University of Illinois Urbana-Champaign, UNITED STATES OF AMERICA

## Abstract

The shortage of healthcare professionals increasingly challenges the provision of care for institutionalised older adults. This study investigates healthcare professionals’ expectations regarding the added value of social robots in daily care, as their perspective is adamant for implementation of these robots but not yet fully investigated. Two consecutive focus group sessions were conducted across three nursing homes with 24 healthcare professionals. The first session focused on identifying potential ways in which social robots could add value for both staff and residents. After sharing suggestions across all groups, the second session facilitated cross-validation and in-depth discussion of practical implications. Data were analysed using qualitative thematic analysis. Healthcare professionals expected several benefits from using a social robot, including reduced workload and mental strain, improved work atmosphere, and potentially heightened job satisfaction. For residents, expected benefits included promotion of self-care and self-reliance through reminders and notifications, and provision of companionship during lonely periods. Reported possible barriers included limited technical knowledge and lack of support from residents’ families. Participants expected that social robots could help save time and energy, enabling more focused attention on residents needing support—even those who may not actively seek it. Professionals indicated that this might enhance both residents’ well-being and the quality and satisfaction of their own work. Implementing some of the specific suggestions from professionals merits further investigation.

## Introduction

In the coming decades, the group of older adults in need of care is growing rapidly [1–3], while the number of healthcare professionals is not increasing at the same rate. As a result, the already existing shortage of professionals that care for older adults is expected to become even more pronounced in the near future [4]. This poses substantial challenges to society at large, as it threatens the quality of care for and life of older adults, and to healthcare professionals specifically by causing an increase in workload and a potential threat to job satisfaction. One of the main problems in the care for older adults is that healthcare professionals often lack the time to provide more than just essential, basic care. Policy makers and designers hope that socially assistive robots (SARs, hereafter called ‘social robots’) can assume some of these basic, formal aspects of care, thereby allowing healthcare professionals to focus more on the human side of their role [5].The expectations and attitudes of front- line professionals toward the use of robots are recognised as crucial for successful implementation; yet, this aspect remains insufficiently understood. The current study uniquely examines healthcare professionals’ expectations of the possible impact of a social robot on their own daily work, along with that on the older adults’ daily life.

Social robots come in all shapes and sizes, varying from pets, such as seal babies or dogs, to fully humanoid service robots [6]. Numerous experiments and studies have been performed using social robots in the care for older adults [7,8]. Most of this research to date has been conducted with the primary aim of assessing benefits such as reducing negative emotions and enhancing social engagement in older adults [9]. Several studies show that in general, different types of social robots are accepted by residents of nursing homes, and that social robots might even play a role in slowing down the progress of cognitive impairment [10]. Previous research indicates that older adults find interaction with the robot quite enjoyable, but consider it a pleasant bonus, an addition rather than a replacement for traditional daily care [11]. On the other hand, some studies suggest that healthcare professionals do expect that a robot could take over or support certain aspects of their task [5] and reduce their workload if they were deployed adequately [12,13]. One study, examining opinions of health care professionals after deploying a social robot in the care for older adults, showed a mutually beneficial effect for healthcare professionals and residents of a nursing home [14]. Specifically, healthcare professionals enjoyed their work more and conveyed this feeling to the residents, while the residents were more prepared to cooperate and thereby made the professionals’ job easier.

However, doubts and concerns regarding the use of social robots in care for older people can lead to resistance among healthcare professionals [15]. Positive expectations about improved efficiency are often accompanied by visions of fewer available jobs and the fear of losing their jobs [5,16,17]. Although robots are often deployed with the purpose of reducing workload and mental strain, they sometimes have the opposite effect [18], or at least the professionals expected so [10]. However, as recent research has shown, in developed economies, the use of robots does not necessarily lead to a decrease in employment [19].

There is still little known about healthcare professionals’ perspectives on how the use of social robots might impact their own daily work. In their scoping review on the (potential) barriers and facilitators to implementing social robots for the benefit of older adults in nursing homes, Koh et al. conclude that a disproportionate large body of research has focused on the characteristics of social robots (internal validity) [8]. In contrast, relatively little attention has been given to contextual factors, such as the attitudes of potential users and the practical implications for professionals of the use of robots in the care for older adults (external validity). Only a small portion of the existing studies explicitly addresses the perspective of healthcare professionals with respect to the implementation of social robots. For the successful implementation of social robots in care for older adults, it is essential to not only understand the expectations, wishes and needs of residents and designers, but also those of the healthcare professionals. Additionally, it is important to deepen our understanding of the specific situations in which social robots could support healthcare professionals in their daily work. Accordingly, this study aims to explore what healthcare professionals expect to be the added value of using a social robot to support their daily work in the care for older adults.

## Materials and methods

### Design

We conducted a qualitative study using focus group discussions (FGDs) with healthcare professionals to explore their expectations of the added value of social robots in their daily work. Focus group discussions are a suitable method since little is known about how healthcare professionals view the possible use of social robots to benefit themselves. By collecting data in groups, a range of issues can be identified with a diversity of suggestions, while extreme ideas can be tempered.

The *Consolidated Criteria for Reporting Qualitative Research* (COREQ) [20] was used as a reporting guide for this article.

### Locations and participants

We recruited healthcare professionals having direct contact with residents at three locations (B, R, and K) of nursing homes in the northern Netherlands. Locations B and R are part of a large institution and offer groups living for older adults with mental challenges, such as dementia, and independent living with care for people with a mild form of dementia or a physical disability. At these locations, different kinds of care are offered, depending on the current physical or mental health state of the residents. Therefore, a division is made into four care sections: older adults living more or less independently, needing some care (home care, HC), people living internally, needing care for physical reasons (somatics, S), people with mild to moderate dementia symptoms (psychogeriatrics, PG), and people in an advanced stage of dementia, actually living together in a group (small-scale living, SSL). Location K is smaller and offers care for older adults in any of the abovementioned stages of retrogression, without being strictly divided into different sections, except for a separate home care section.

At each of the three locations, a focus group of healthcare professionals was formed. The recruitment took place through general announcements in the form of posters, flyers, and group emails at the care facilities. The sample was based on convenience, and the professionals participated voluntarily. In total, 24 healthcare professionals participated; some of their characteristics are described in Table 1.

**Table 1:** Characteristics of the participating healthcare professionals (n = 24).

Code	Gender	Age (years)	Education^1^	Job description^2^	Years in function	Section^3^
R101	m	56	MBO	Nurse	5	SSL
R102	f	27	WO	Psychologist	0.5	All sections
R103	f	26	WO	Psychologist	4	All sections
R104	f	31	HBO	Quality Nurse	1.5	SSL
R105	m	27	HBO	District Nurse	2	HC
R106	f	49	MBO	Residential Care Assistant	3.5	All sections
R107	f	missing	HBO	–	–	All sections
R108	f	27	MBO	Nurse	3	HC
R109	f	25	MBO	Nurse	5	HC
R110	f	26	WO	Psychologist	3.5	All sections
B201	f	52	MBO	PHA	3	S
B202	f	53	MBO	PHA	30	SSL
B203	f	25	MBO	PHA	3	PG
B204	f	39	MBO	PHA	2	PG
B205	f	20	MBO	PHA	0.5	PG
B206	f	37	MBO	PHA	7	S
B207	m	29	MBO	PHA	3	S
K301	f	48	MBO	Activity supervisor	2	S, PG
K302	f	24	MBO	PHA	4.5	S, PG
K303	f	22	MBO	Nurse (intern)	1	S, PG
K304	m	37	HBO	Nurse	16	S, PG
K305	f	43	MBO	Nurse	1	HC
K306	f	48	MBO	PHA	0.5	S, PG
K307	f	21	MBO	Nurse	0.5	HC

^1^*MBO – Secondary vocational education; HBO – Higher professional education; WO – University education*.

^2^*PHA - personal healthcare assistant (‘Verzorgende IG’)*.

^3^*Care sections professionals are (mainly) active in: HC – home care; S – somatics; PG – psychogeriatry; SSL – small-scale living*.

*R105 and B203 were present only during the first session, and R110, B207, and K307 were present only during the second session.*

### Ethics

The recruitment started Sept 8, 2022. On Sept 29, 2022, the recruitment was completed. Prior to their participation in the focus group discussions, the participants read the information letter, completed a short questionnaire with their basic personal details (position, age, gender, years in this job), and signed the informed consent form. The Ethics Committee of Hanze University of Applied Sciences Groningen (HEAC) approved the research on Sept 6, 2022 (heac.2022.026).

### Data collection

All three focus groups had two meetings. The preliminary findings of the first meetings, which were based on data from all the groups, were shared in the second meetings to deepen the participants’ view on possible applications of the robot and to avoid important suggestions to be overlooked. The discussions were moderated by the primary researcher, who is a senior lecturer at the School of Social Studies and is trained and experienced in qualitative research methods. She had no professional or personal relationships with any of the participants at the beginning of this research. In each session, the moderator introduced herself and informed the participants about the goal and the relevance of this research. During the focus group discussions, the researcher was assisted by an assistant moderator with a bachelor’s degree in communication and multimedia design. Each focus group discussion lasted between 80 and 100 minutes. To make verbatim transcriptions, audio recordings were made of all the discussions. A video recording was also made, exclusively as a backup. During all the sessions, the moderator and the assistant moderator took field notes. The questions and prompts used in the focus groups were prepared and provided by the moderator. The questions asked in both sessions are shown in Table 2. A try-out session took place with a group of PhD students and postdoc researchers from the Qualitative Research Group of the Department of Health Psychology.

**Table 2:** Questions and assignments in the FGDs.

Session 1	
Question	
A	*Now take a few minutes to mentally run through an average day and write down all the interactions and activities with the residents you encounter. You can keep these questions in mind:*
	*- What truly needs to be done every time?* *- What do you often run into?* *- What takes a lot of time and energy?* *After this assignment, the presentation about robots and introduction to Maatje took place*

B	*Do you have any experience with using a robot or have you heard about it in your work?*
C	*Now, use your notes from the first question and write down in what aspects of your work you expect Maatje to have added value.* *According to where the discussion went, additional questions were asked, such as:* *- What exactly do you do and on what occasions would you have liked to use a social robot because you did not get the chance to do it yourself?* *- What could a robot mean for you in your work? Where would you like to deploy a robot in order for it to assist you in your daily tasks?* *- Where would you like to deploy it to help the resident and/or yourself?* *- In what way could a robot help you to make your work a bit more pleasant, more valuable or maybe a bit easier?* *- How would you like to deploy it? This includes fun things you would like to do, but for some reason, are not able to do.* *- What are things that keep happening that make you think: ‘This again? There must be a better way to solve this.’, or things that take up a lot of your time and keep happening? Which actions are often repeated and where do you think it could be useful to use a robot?*
**Session 2**	
D (first list)	*From this list, select two items that you think will be most useful to lower the workload in your daily practice. If you think of some other items which are not on the list, you can add them and we can discuss them too*
E (second list)	*From this list, select two items that you think will be most useful to enhance your work pleasure and job satisfaction in your daily practice. Also here, please add if you miss an important item*

#### Protocol session 1.

After the welcome, a short explanation of the procedure was given. The participants, the moderator and the assistant moderator introduced themselves. For the first assignment, the participants were asked to take a few minutes to mentally run through an average day and write down the most important interactions and activities with the residents they encounter: what truly needs to be done every time, what do they often run into, and what takes a lot of time and energy (Question A). After their answers were written, the participants were asked to keep them for later discussion.

Subsequently, the moderator gave a presentation about the history of robots, the state of the art in robotics, and the typical features of the social robot Maatje*.* This was done to convince them that the use of the robot might truly help them, and working with it is quite easy. Several “Maatje” robots were on hand, allowing participants to examine them up close, pick them up, and try them out. By demonstrating the way the robots are already in use, they began to think about the possibilities for their own work. Maatje is a 25 cm tall, white, humanoid robot that can be programmed and fine-tuned to the resident’s wishes or needs by means of an online platform. It can use voice messages such as notifications, announcements, and reminders, start a short dialogue, and ask about the mood. In addition, Maatje can provide amusement by dancing, playing music, telling jokes, or playing games [21].

The presentation was followed by a question about their experiences with robots in their work (Question B). The next step was to use their notes from the first question and write down in what aspects of their work they would expect Maatje to add value (Question C). Each in turn, the participants revealed what they had written, after which an open discussion about the given suggestions followed. During this discussion, the moderator asked additional questions to encourage the participants to brainstorm about possible applications of the robot Maatje to support them in their daily work. Eventually, the moderator gave a brief description of how everything that was said during this session would be processed.

Between the first and second sessions, the recordings were transcribed verbatim. With these transcripts, preliminary coding was performed to facilitate the discussion in the second session. The preliminary codes were summarised and separated into two preliminary themes: one with codes on how to restrict or lower the workload and the other with codes to increase work pleasure and job satisfaction.

#### Protocol session 2.

In this second session, the moderator handed out two lists, composed of the preliminary findings from the first sessions of all three focus groups. As a result, cross-validation took place, so that the findings of the respective groups could complement or confirm each other. On the first list, participants were asked to select two possible applications of Maatje that they expected to be the most useful in lowering their workload in their daily practice. If they thought of other items that were not on the list, they could add them, and these would be discussed as well (Table 2, Question D1). Every participant explained their choices, followed by a group discussion. The second session was wrapped up by thanking all the participants and presenting them with a small token of appreciation.

### Data analysis

The inductive thematic analysis (TA) technique was used following the six steps described by Braun & Clarke [22]. After each recorded FGD, verbatim transcription was performed. The assistant moderator checked the transcriptions by listening to random samples of the recorded sessions. The coding of the transcripts on paper was independently performed by both the head researcher (SB) and the secondary coder (MT), a senior researcher trained and experienced in qualitative research methods. Both coders subsequently discussed their results. During the coding, reviewing potential themes, defining themes, and naming themes occurred. Disagreements were solved by discussion and recorded in a logbook. The themes from each of the transcripts were extracted separately, after which the codes and themes were compared and discussed until a final code book was established. Eventually, themes and quotations were translated from Dutch to English for publication.

## Results

**Fig 1:**
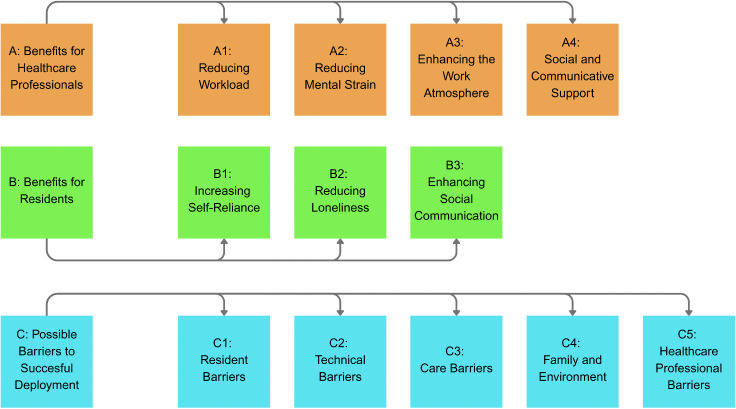
Visualisation of results.

The analysis of the focus group discussions about the expected added value of using a social robot as support in healthcare professionals’ daily work in the care for older adults resulted in three themes: A) expected benefits for healthcare professionals, B) expected benefits for residents, and C) possible barriers to successful deployment of the robot. The themes are described below, and Tables 3–5 present an overview of the fitting quotes for each of the three themes. It is important to note that the context of the professionals’ work plays a role in determining the added value that a robot can yield. How a robot might be deployed will depend on which care section the older adults are residing in. In the discussions, healthcare professionals came up with many suggestions, which were primarily applicable in the setting where they work. However, the main themes emerging from the discussions were the same, independent of context. A visualisation of the main themes and subthemes can be seen in Fig 1.

**Table 3:** Overview of Theme A: Expected benefits for healthcare professionals.

Subthemes briefly summarised	Quotes (respondent number)
**A1 Reduce workload** Care professionals expect the robot to reduce their workload; time saving most frequently mentioned	*Q1 “Maybe a reminder of medication; most residents can take these themselves, but they will just forget to do it. Then, it would be nice if a robot would notify them, that will save us another walk and some precious time” (K303) … “Residents often ask when somebody will come to give medication, it would be useful if the robot could inform they can take it themselves” (K302)* *Q2“Reminders, for example someone has a catheter, which is usually changed every 6 weeks and sometimes a resident asks ‘when does it need to be changed again, if that is entered in Maatje (‘on this date it has been replaced and then and then it has to be weather’). The resident can then ask ‘when do I need a new catheter’ and the robot can also answer that question... then you can let go a little bit, well, of course you cannot let go completely because you ultimately have to do it yourself. It surely saves us time because otherwise they would ask us all the time, then you have to go back to the computer to look it up, then back to the resident and the next day the resident has forgotten it and he asks the same question again, do we have to start the same thing again, so if he can ask the robot, then that is a definite time gain’ (K304)* *Q3 “Can we also set something to ensure that they do not switch day and night? This happens quite often, that they sleep a lot during the day and are awake at night; Can you set it so that it wakes someone up every so often?” (B202)… “We have just moved someone who had the same problem, walking across the corridor at night. It is nice when the robot says it is the middle of the night, let us go back to sleep. Then, we do not have to walk there all the time” (R105)*
**A2 Reduce mental strain** A robot may reduce stress by answering some repeatedly asked questions	*Q4 “You actually walk back and forth constantly, often you are doing an activity with someone or you just want to start it and then you get another call from someone else, you have to go there again” (B203)* *Q5 “If you see during the break between 12 and 1 that a resident calls continuously because he always has a few* *questions; then it is your moment of rest, if in that situation you are simply disturbed by questions that do not truly matter, then you also want to be able to think ‘* *not now, now I want some time for myself’* *. (K304)* *Q6 “One resident always calls for the same thing; it would be useful to be able to send a message, but also helping people who use the bell alarm excessively, which is sometimes very inconvenient, suppose you are dealing with someone who really needs an ambulance, then you are just so busy that if someone is constantly there If you are calling with questions such as when are we going to eat or what time is it, you simply do not have time for that. The other person then has priority, but a resident who constantly forgets things obviously cannot empathise with that. Yes, then such a buddy might be very useful who will then say ‘so and so late’, so that the pressure disappears, so that you can just do your thing (K302)*
**A3 Work atmosphere, job satisfaction** A robot may help professionals to divide their time between more and less demanding residents	*Q7 “If someone is happy because they have just listened to ‘Tulips from Amsterdam’, then we come in there and then you start the conversation very differently than if someone says ‘hmm it is raining outside again…’, so you go inside more pleasantly, also have a better state of mind and can move on to the next resident with a smile and eventually go home with a good feeling … It also has to do with the scheduling of staff, of course if your schedule is tight and you do get around to the work for which you are actually scheduled as staff, then there is no problem if there is no further unplanned care; if you could accommodate that with Maatje you can also go home with a more satisfied feeling because you have completed your work well” (K304)* *Q8 “Yes, it is sometimes quite difficult, especially if they want to tell whole stories, and you have to move on, then they do not appreciate that; if a robot can play a game with them, so that they feel like they have something to do or something... then I leave with a better feeling and they might hold me or my colleagues a little less... (laughing) Yes, you stand there like ‘* *I actually have to leave’* *but you actually want to listen because yes... and then sometimes you have to break in and say ‘* *yes, I really have to move on now’* *(K305)* *Q9 “Some people are very independent and hardly ever ask anything; it is nice if they also get attention” (K302)*
**A4 Social and communicative support** A robot may release professionals from ‘keeping the conversation going’	*Q10 “When people become more and more apathetic, it is difficult to have a conversation together, so it is more that Maatje can provide some kind of distraction, that kind of thing, instead of having a conversation together.” (R101)* *Q11 “I also think of small-scale living, simply as a means of activation … if you, as a professional, do not know how to start a conversation at the kitchen table, for example” (R107)*

**Table 4:** Overview Theme B: Expected benefits for residents.

Subthemes briefly summarised	Quotes (respondent number)
**B1. Increasing self-reliance in the resident** Reminders might make residents feel more confident about their abilities	*Q12 The resident can ask ‘when do I need a new catheter’ and then the robot will answer that question … this gives the resident a certain peace of mind, so that you even could receive a reminder from the resident ‘I will get a new catheter on 15 December’, which also gives the resident more control over his care process and I think that a bit of self-direction is an important thing.”(K304)* *Q13 “And number two (reminders for activities) is also very important for our residents (R105)... “we are not allowed to take them to the Bingo for example, we are not allowed to schedule a care moment for that, so the robot would be ideal for that” (R108) … “yes, because if we come to someone at 7 o’* *clock in the morning we can say yes, Bingo is at half past two, but by then they have forgotten that again, so if they can receive a signal from the robot fifteen minutes or half an hour in advance saying ‘* *you can put on your coat, you are going to the brasserie’* *, that would be fantastic for certain residents of ours” (R105)*
**B2 Reducing loneliness** A robot might provide a sense of companionship.	*Q14 “Why does someone feel lonely? Because he likes attention, that is why people often press the bell with a question. When the question is answered they feel they are being heard even if it is by a robot” (K304)* *Q15 “Sometimes it can also be quiet for a long time in such a room; it would be nice if the resident occasionally hears something that is personal, so that he knows that he is being thought of … It is good to prevent them from becoming lonely, so that they do not feel like they have just been brought here, that no one cares about them anymore and that they can die without anyone caring. A robot could also be a kind of gateway to a larger area than just life here. For example, the robot could play back stories from the past recorded by one of the children”. (K304)* *Q16 “It is important that the resident feels heard, that is important to prevent the feeling of loneliness” (K303)* *Q17 “The robot can also be something you can just talk to.” (R101)* *Q18 “Maatje could make music then, and do a nice dance, maybe that will make people laugh”(R104*
**B3 Enhancing social communication**	*Q19 “Could he play a game too? I would really like to play games, we have a nice set of cards with questions about the past, people appreciate that; it would be nice if Maatje could do that with the people” (B202)* *Q20 “Maatje looks like a human and the point is to make a connection, right? I can imagine that a personal connection could develop” (R101)* *Q21 “I think … at home care people might be able to have a conversation with the robot and at small-scale living it is more likely for the robot to break the silence or answer questions” (R102)*

**Table 5:** Overview Theme C: possible barriers to successful deployment of a robot.

Subthemes briefly summarised	Quotes (respondent number)
**C1 Resident Barriers** Functionalities should be fitting to residents’ mental and physical condition	*Q22 “Yes, in theory it is nice, but you’re dealing with people with severe dementia and they very often do not understand something you say, or take it differently, so how do you deal with that, even if Maatje says something” (R103)* *Q23 “There are people who can stand only little stimulation” (B202)*
**C2 Technical barriers** Concerns about technical problems may form a barrier for using a robot.	*Q24 “... during the pilot I saw that the functioning of Maatje was not always perfect… a carer came to say ‘it does not work again’ or ‘the battery is empty again’ … just the pure technology, that truly has to be working for 100%” (R107)*
**C3 Care barriers** Robot not useful in all situations	*Q25 “I also think it’s tricky with alarming behaviour, because if someone is lying on the floor and the robot says ‘we will be back later’, that is not what you want either” (R104)*
**C4 Family and environment barriers** Family should be cooperating	*Q26 “we have someone who has poor eyesight but truly likes books and if someone reads in a pleasant voice that would be nice for her (B201)”...”we have a resident who also has that and the family has purchased an e-reader for this purpose, we then turn it on, the resident puts in earphones and sits down to listen; yes, that is just how that family is”… “not every family is that far along” (B202)*
**C5 Healthcare professional barriers** Insufficient technical knowledge can lead to uncertainty or resistance in the professionals. Adequate training is important. Also possible ethical objections.	*Q27 “I have not been interested in technology and domotics for very long,.. I’m not very familiar with it yet …I took a course online, but I could not apply it..., I lost that knowledge again … you learn the most in practice” (B201)* *Q28 “It would be best if we received training here at <location> like now, during consultation time... and immediately get started with the robot to try it out … Yes, then you immediately know how it works … with a short explanation next to it, max 1 A4, in case you forget something and for colleagues who are not here”(B201, 202)* *Q29 “* *I think they’d prefer to have a human being facing them than a robot”* *(K301)*

### A. Expected benefits for healthcare professionals

The well-being of healthcare professionals is sometimes under pressure. They would feel better and more connected to the residents if they could pay more timely attention to the residents who need it and offer them the quality they would like to give. At times, they even skip breaks or other moments of rest, for example, when colleagues are absent due to illness. Some relief through the deployment of a robot might, in the professionals’ view, contribute to their well-being. The data analysis of all the discussions yielded four subthemes: reducing workload, reducing mental strain, enhancing the work atmosphere and job satisfaction, and providing social and communicative support. The relevant quotes for these subthemes are shown in Table 3.

#### A1. Reducing workload.

The findings showed that all the participating healthcare professionals expected the robot to be able to reduce their workload in various situations. While this was a general feeling, there are differences, depending on the intensity of the care and the way it is arranged. Time saving was the most important item in all care sections. Healthcare professionals must walk to the residents’ apartments quite often to remind the residents to perform actions they can do themselves, resulting in a high workload. People with mild cognitive impairment or loss of memory (home care, psychogeriatrics) could be reminded by the robot to take their medication, or the robot could inform them about coffee time or social activities. Residents who are forgetful can also be fearful of forgetting things that are planned and therefore keep asking about them. A robot might be able to answer many of these questions. The professionals indicated that they sometimes skip their planned break. In their view, a robot might offer a way to prevent this by filling the gap of a missing colleague during breaks. That is, if the professional goes on a break or is called away for unplanned care, the robot might keep the residents company, entertain them for a while or be ready to answer questions.

#### A2. Reducing mental strain.

Especially in the small-scale living care sections, people tend to forget what they asked and what the answer was; hence, they are asking the same things repeatedly. In the long run, this places considerable mental strain on healthcare professionals. They expect significant benefits if the robot were able to answer (part of) these questions. In most cases, residents push a call button to request help, and the professionals are expected to respond to such a call for help even if they know it is not urgent. As such, it can be very stressful mentally if they keep being called away while they are engaged in an activity that needs all their attention or manpower.

#### A3. Enhancing the work atmosphere and increasing job satisfaction.

Healthcare professionals in the different care sections expected the robot to be able to enhance the work atmosphere and give them more fulfilment in their jobs in various situations. They indicated that almost all the time available is spent on residents who need something or have questions; there is hardly any time left for residents who demand little or no attention. The professionals expect to have a sense of peace if a robot could help fill in the gaps of time for these undemanding residents. Alternatively, a robot might partially take care of answering repeatedly asked questions as already suggested above. Similarly, in the small-scale living section, it was mentioned that a robot might entertain the residents by playing a game with them or dancing or playing music. It is expected that in addition to the residents, the healthcare professionals may also become better humoured. Another possibility for increasing their job satisfaction might be that a robot announces the professionals’ regular visits, for example, to help them get dressed. If the residents were pre-reminded about this, they might look for their clothes and put them ready in advance. Such a cooperative attitude would positively influence the professionals’ mood, generating a pleasant entrance and providing space for informal chat.

#### A4. Social and communicative support.

In sections where residents suffer more and more dementia or forgetfulness, communication can become difficult. The daily conversations will regularly fall silent. For healthcare professionals, it is rather frustrating to fill these gaps all the time, and they envision a role for a social robot in keeping the conversation going and making it more pleasant.

### B. Expected benefits for residents

The findings showed that in the eyes of healthcare professionals at all locations, Maatje might be able to contribute to the wellbeing of residents in various ways. The data analysis of the three focus group discussions revealed three subthemes related to benefits for the residents: increasing their self-reliance, reducing loneliness and enhancing social communication. The relevant quotes for these subthemes are shown in Table 4.

#### B1. Increasing self-reliance.

In the home care sections, residents are stimulated to rely on their own abilities to take care of themselves for as long as possible. Healthcare professionals indicated that while residents can still perform several actions independently, they often forget to do them. It might give them a feeling of confidence if a robot would remind them and give notifications of what will happen next or what needs to be done: a reminder to take medication, a notification of activities such as bingo or coffee, or a notification that a healthcare professional or visitor is arriving. If the robot could offer help in maintaining the right day‒night rhythm, this might also stimulate residents to participate in activities.

#### B2. Reducing loneliness.

Residents are sometimes lonely, especially in the hours during which a resident is alone in the apartment. A robot might help by giving them a sense of companionship. Several possible actions were mentioned: answering questions, asking a personal question from time to time or reminding the resident of the hygiene protocol. Furthermore, a robot might offer some entertainment by reading a book or newspaper aloud, playing music, or even performing a dance act.

#### B3. Enhancing social communication.

In the eyes of healthcare professionals, it is important that residents maintain a certain level of social interaction. A robot might be able to offer help in several ways: it could make small conversations with quiet or nondemanding residents or ‘kill the time’ by playing games with the residents. It was also suggested that residents can build an emotional connection with the robot. Maatje’s friendly appearance might promote such a connection. In the small-scale living sections, professionals also see possibilities for a social robot to calm down residents in the case of restlessness or restless sleep.

### C. Possible barriers to successful deployment of a robot

In addition to benefits, the healthcare professionals mentioned some barriers they expected. The data analysis of all the discussions yielded five subthemes: resident barriers, technical barriers, care barriers, family and environment barriers, and healthcare professional knowledge barriers. The relevant quotes for these subthemes are shown in Table 5.

#### C1. Resident barriers.

Depending on the age-related disease they suffer from, some features of the robot are not very useful. For example, if people cannot understand a robot due to their mental state, it would have no effect or even lead to a dismissive attitude. People might also be hypersensitive to unexpected stimuli; this may make a robot uttering unannounced messages unfit for them. Professionals indicated the importance of adapting the functionality to the individual residents; otherwise, it might miss its goal.

#### C2. Technical barriers.

Concerns about technical problems can form a barrier for using a robot. The participating professionals indicated that the robot must perform optimally on a good working WiFi network. In addition, it was mentioned that it must be kept hygienic and should always be sufficiently charged.

#### C3. Care barriers.

In some cases, healthcare professionals had doubts about whether a robot could truly take over a task. For example, if the alarm call button is used frequently, you can never be sure that there is no emergency. When care is organised in groups, as in the small-scale living sections, the wishes and needs of residents can differ so that one robot cannot serve everyone.

#### C4. Family and environment.

A dismissive attitude from family and other close relatives might form a barrier. Some residents have family members who introduce technical aid themselves, such as an e-reader that can read aloud books for their visually impaired relative, but not every family is ready for such innovations. The professionals state that for a successful deployment of a robot, it is important that the family/informal caregiver is involved.

#### C5. Healthcare professional barriers.

Insufficient technical knowledge might lead to uncertainty or resistance in the healthcare professionals to take the robot into use. They emphasise the importance of adequate training, preferably on the work floor. Professionals may also have ethical objections, such as the presumed ‘inhumanity’ of a robot.

## Discussion

We explored what healthcare professionals working in a nursing home expect to be the added value of using a social robot to support their daily work. When we look at the expected benefits for the professionals themselves, two main issues were identified: they expected that the robot might reduce their workload and increase their job satisfaction ⎯ not only due to the anticipated (expected) reduction in workload, but also because it might improve the quality of life for the residents in their care. These expectations might affect real-world implementation. For example, if it were found during implementation that the social robot could indeed reduce the workload of healthcare professionals, this might ensure sustainable integration of the robot into practice. If the robot could also increase their job satisfaction, this might lead to lower turnover and, consequently, to an additional reduction of workload. Thus, the combination of these two expected advantages might initiate an upwards spiral.

The professionals also mentioned a substantial number of situations in which the social robot ‘Maatje’ might contribute to improvements in the residents’ lives. Using these situations might increase the chances of successful and sustainable implementation. If the robot were able to help residents: (1) remember important activities or appointments, (2) provide distraction during moments of inactivity, and (3) promote self-care and personal hygiene, this would be advantageous to the professionals in terms of workload and benefit the residents themselves as well. Also, if residents would become more self-reliant or would generally be in a better mood, the professionals’ job satisfaction might further increase.

Our study adds significantly to the understanding of wishes and expectations of healthcare professionals working with older adults concerning the use of a social robot to support them in their daily work. Other studies (e.g., [9]) examined residents’ needs or were designer- or researcher focussed. By focusing fully on the professionals’ views, we acquired vivid and detailed information about how healthcare professionals picture situations in which a robot truly could have added value for them and the residents in their care. This information may be useful, or even indispensable, if it comes to real-world implementation. The health care professionals are the bridge between the organisation on one hand, and the residents and their informal caregivers on the other hand. Trying to deploy robots where the professionals do not expect these to be useful will be a hopeless spending of time and money; it will probably lead to the robots ending up somewhere in a junk room or closet. Therefore, it is wise to involve the professionals in implementing the technology and introducing it to the residents.

The professionals in our study make it clear in their statements that for them, the residents’ interests are paramount. They are intrinsically motivated to improve the residents’ quality of life. If a robot can contribute to this, it will give them more job satisfaction. This employee attitude is also highlighted as a facilitator for implementation in an other qualitative study [23]. It also emphasizes the importance of involving stakeholders, such as healthcare professionals and family members, a finding which is echoed in our study as well. The professionals in our study indicated that, on one hand, for a successful deployment of a robot, it is important that the family/informal caregiver is involved. On the other hand, they point out that a dismissive attitude from family and other close relatives might form a barrier. While some residents have family members who even introduce technical aid themselves, not every family is ready for such innovations. Previous research revealed that informal caregivers can play an important role in determining and communicating the residents’ wishes and needs to responsible professionals [24]. In our view, the programming of the robot with fitting notifications and reminders might become an act of co-creation between healthcare professionals, residents, and their informal caregivers. Further research into the latters’ role would be worthwhile; in the end, they are the ones who know the residents best and therefore should be able to play a significant role in fine-tuning the robot to the needs of their institutionalised partners or parents.

Healthcare professionals in our study mentioned sufficient available time, digital competence, and adequate training as conditions for a successful incorporation of a social robot in their daily work. This corresponds with results of a recent study in which a social robot was deployed in practice in a group of people with intellectual disabilities [25]. Professionals were inclined to keep using the device, if they had enough available time and resources, and training. Considering the issue of time, it is worthwhile noting that there is a tension between the expectation of saving time and the need for more time. In our study, it was clearly stated that a significant investment of time would be needed to get used to a robot, to receive training and to program the right information into the robots. After this ‘warming up’ period, time might be saved, compared to the current situation by using the robot in situations suggested by the professionals. The results of another longitudinal study were less optimistic regarding saving time, but it did report a different and more favourable use of the available time [26]. Specifically, as the social robot was deployed for daily, routine actions, there appeared to emerge a better one-to-one communication between healthcare professionals and residents. This corresponds with the wish and expectation of the professionals in our study to have more time for personal contact with the residents.

The professionals in our study seemed very motivated to start using a robot and to share their expectant thoughts about this. Possibly, this positive attitude was caused by an interactive session with the robot, which was held at the beginning of the project. In a recent study, showing that the level of intention to use robots in health care was moderate among healthcare professionals, this physical confrontation did not take place [27]. This example shows the importance, as predicted by the Technology Acceptance Model (TAM), of giving future users a real-time experience with new technology. TAM states that the intention to use new technology is most significantly influenced by ‘perceived usefulness’ and ‘perceived ease of use’ [28]. Although our study did not directly measure the intention to use a robot, the participants’ attitudes towards a robot were mainly positive. However, it is possible that this positivity was influenced by our recruitment procedure, which may have attracted individuals who were already interested in or open to the technology.

In our study, improving or maintaining social and cognitive skills in older adults with Mild Cognitive Impairment (MCI), e.g., by asking and answering simple questions or playing games, was seen as one of the possible benefits of the use of social robots. This aligns with the studies in a scoping review, confirming the promising role of socially assistive robotics in interventions for mild cognitive impairment [10]. The authors of this study advocate for increased collaboration between clinicians and robotics researchers to develop interventions targeting social cognitive skills. It is important to conduct further research in the future into the effect of cognitive and social interventions with social robots, so that they can be used to slow down the development of more severe forms of cognitive decline.

Regarding possible barriers to implementing robots in health care, the technical barriers mentioned in our study were primarily practical problems and conditions: the robot must perform optimally, the Wi-Fi network must work properly, and the robot must always be sufficiently charged. This is consistent with a study that showed practical difficulties to be a limiting factor [29]. In the contexts studied there, practical difficulties were also considered a barrier. Furthermore, our participants mentioned lack of technical knowledge as a possible barrier for robot use, which was also found in [23]. According to participants in our study, insufficient technical knowledge can lead to uncertainty or resistance in healthcare professionals to take the robot into use. They emphasise the importance of adequate training, preferably on the work floor.

An ethical issue in our study was the possibility, or even probability of a personal connection between the resident and the robot. However, this was not reported as a possible barrier; rather, it was seen as a positive aspect. While in literature with normative arguments about use of technology the concept of companionship and attachment also has negative connotations, research shows that professionals on the work floor are mainly positive [30]. The health care professionals in our study thought that feeling connections, even with a robot, is better than not feeling any connection at all. They expected that the residents would feel heard, even if it were by a robot. Especially in times of long-lasting isolation, a social robot might provide a suitable alternative for actual company. It has been reported that during the COVID-19 pandemic, social robots were perceived as more useful than before [31]. As real visits often were not possible, an attempt was made to replace these by using digital and robotic interventions. This period accelerated the digital transformation of many organisations, thereby increasing the use of robots [32].

A study, performed during the pandemic in Canada [33] did reveal some ethical issues. For example, while social robots emerged as very useful in those days of reduced contact, at the same time the concern was raised that the prolonged absence of visitors might be accepted too easily because ‘the robot is already there’. In our study, the importance of the family’s commitment and involvement in the deployment of a robot was mentioned as a significant factor. We believe that if the family is involved from the outset, it is unlikely that their frequency of visits would decrease if a robot were placed in the room of their beloved. However, further research is needed to confirm this assumption.

The current research has some strengths and limitations that must be kept in mind when interpreting the results.

One potential limitation could be the influence of group dynamics inherent to the focus group method. A drawback of the joint discussion of expectations, wishes, and needs is that participants may have withheld suggestions that differed greatly from the main consensus [34]. However, the group discussion also created opportunities for participants to inspire one another and collaboratively develop optimal choices and ideas.

A second limitation concerns the recruitment strategy. Since the recruitment was based on voluntary participation and availability, the sample cannot be taken to be representative of the whole population of healthcare professionals in the care for older adults. The participants did come from a range of care sections, which yielded a certain level of representativeness.

A third limitation might be the prior exposure of the robot to the participants. Before the focus group discussions started, the head researcher gave a demonstration about robot technology in general and the proposed social robot Maatje in particular. The participants may be slightly biased after seeing this and their imagination may have been limited. However, we thought it was useful to give at least a first impression about the possibilities and restrictions of the robot.

A fourth limitation pertains to the coding process that took place after the first session. Between the two sessions in each group, only preliminary coding of the first session could be performed. That is, after the transcriptions of all the first sessions were read, a short list was set up and handed out to the participants to deepen the discussion of the second sessions and to add cross-validation to it. Consequently, we may not have been able to bring in everything that was said in the first sessions. However, this was a deliberate choice to strike a balance between reiterating enough information and not making the period between conversations too long. Additionally, it was emphasised during the second session that the participants could bring new solutions or items that were not previously mentioned into the discussion, which resulted in even more comprehensive information.

The current study provides several practical implications for nursing homes that want to implement social robots. First, developing on-site training programs for healthcare professionals is crucial; the participants emphasized the importance of adequate education and training on the job for using the robot. Due to the high turnover in nursing home personnel, it is important that training is offered systematically and regularly on the work floor. A short user manual should also be placed near the robot so that healthcare professionals can check it when needed. Second, co-design sessions with families and training for informal caregivers should be organized; family involvement may reduce the workload of formal caregivers and improve the sustainability of the adoption. Third, it is important to facilitate and support healthcare professionals by providing enough time and resources to use and maintain the robot. The professionals in our study expected the robot to save them time; however, in the beginning, an extra time investment will be necessary to get used to the robot. Fourth, the care professionals should determine, together with the residents and their informal caregivers, who may benefit from a robot and with which purpose (e.g., improving self care, especially oral hygiene, or reminding residents to drink enough). As the professionals indicated, not all residents are ready and open to using a robot. Taking these recommendations into account may facilitate the implementation of a social robot into the daily care for older adults.

## Conclusions

Healthcare professionals often have clear perspectives about what is needed to improve working and living in a nursing home. The professionals in this study felt that if robots were deployed, they might save time and energy to pay attention to the residents who need it, even those who do not ask for it. Not only did the healthcare professionals expect this to enhance their own job satisfaction, but the well-being of the older adults as well. Based on these positive expectations, it is certainly worthwhile to find a way to put their concrete suggestions, such as attention to self-hygiene, the involvement of informal caregivers and training on the floor, into practice. This might positively affect real world implementation.
